# A disinhibitory microcircuit in the temporal association cortex for fear retrieval to pure tones

**DOI:** 10.1038/s41598-025-05566-0

**Published:** 2025-07-01

**Authors:** Rui Cheng, Wen Zhong, Yangqiu Yan, Linhui Yao, Peiran Yin, Ziyi Xu, Xiaoxia Qin, Jie Tan, Yingying Zeng, Jinhua Liu, Zhongju Xiao

**Affiliations:** 1 Department of Physiology, School of Basic Medical Sciences, Key Laboratory of Psychiatric Disorders of Guangdong Province, Guangdong-Hong Kong-Macao Greater Bay Area Center for Brain Science and Brain-Inspired Intelligence, Key Laboratory of Mental Health of the Ministry of Education, Southern Medical University, Guangzhou, 510515 Guangdong China; 2 School of Traditional Chinese Medicine, Guangdong Basic Research Center of Excellence for Integrated Traditional and Western Medicine for Qingzhi Diseases, Southern Medical University, Guangzhou, 510515 Guangdong China; 3 The Seventh Affiliated Hospital, Southern Medical University, Foshan, 528244 Guangdong China

**Keywords:** Fear retrieval, Temporal association cortex, Tail of striatum, Auditory, Disinhibition, Learning and memory, Neural circuits, Neuronal physiology

## Abstract

Fear retrieval is fundamental for animals to rapidly enter a self-defense state, which enhances their survival rate. Although the cellular mechanisms and neural circuits underlying fear retrieval have been relatively well studied, particularly in classic fear-related nuclei such as the amygdala, the neural circuits and intrinsic microcircuits of some non-classical fear-related nuclei remain poorly understood. A disinhibitory microcircuit in the temporal association cortex (TeA) was identified in this study, demonstrating a critical role in fear retrieval. Additionally, the response of the TeA to fear retrieval is largely driven by acetylcholine (Ach), which then relay information to the tail of striatum (TS) to mediate freezing behavior. Together, these findings demonstrate the essential role of the TeA and its microcircuit in auditory fear retrieval in response to pure tones, providing new insights into the mechanisms of fear retrieval in the cortex.

**Supplementary Information:**

The online version contains supplementary material available at 10.1038/s41598-025-05566-0.

## Introduction

Sensory inputs must be processed by neural circuits in the brain to trigger corresponding behaviors. Auditory fear conditioning is a classic form of associative learning, acquired through the temporal pairing of a neutral stimulus with an aversive stimulus, which elicits a behavioral response known as freezing^1,2^. The amygdala is currently regarded as a key structure mediating fear learning and memory, integrating information about the conditioned stimulus (CS) and unconditioned stimulus (US) through local synaptic plasticity^3–7^. Traditionally, it has been posited that lesions of the auditory cortex (AC) predominantly impact auditory fear conditioning to complex sounds^8^ rather than to pure tones^9,10^. However, with advancements in technology, it has been discovered that the auditory cortex plays distinct roles in different stages and complexities of fear conditioning. Unlike the AC, which regulates fear memory of complex sounds, inhibition of the TeA affects fear memory of all types of sounds^11,12^ reflecting a unique processing mechanism for auditory information in the TeA. Nevertheless, the neural circuits and neuronal types underlying auditory fear conditioning in the TeA remain poorly understood.

The TeA, a nucleus adjacent to the AC, was initially studied as part of the AC^10,13,14^. However, as its anatomical projections were further elucidated^15^ it has been demonstrated to play critical roles in auditory fear conditioning learning and memory^11^ maternal sensitivity to pup calls^16^ and even regulation of NREM sleep^17^. When tracer viruses were employed to delineate the downstream nuclei of the TeA, studies have shown that its primary subcortical projection targets include the TS^18,19^ and the lateral amygdala (LA)^11^. Optogenetic silencing of TeA projections to the LA has been shown to primarily impair fear memory of complex sounds in mice^11^. In addition, the TS, initially associated with reward mechanisms^20–22^ has recently been shown to be closely linked to auditory fear conditioning memory^23–25^. However, the specific functional role of TeA projections to the TS remains unexplored.

To execute precise life activities, the neocortex has evolved a diverse array of GABAergic interneurons^26–33^. Different types of inhibitory interneurons regulate cortical network activity by forming neural circuits, among which disinhibitory microcircuits controlling conditioned fear responses have been studied^34,35^. It has been reported that in the AC, a disinhibitory microcircuit mediated by layer 1 interneurons regulates fear learning^35^. While the TeA is also involved in auditory fear processing, it remains unclear whether similar microcircuits regulating fear processing exist within it.

## Results

### Direct projections from TeA to TS

**Fig. 1:**
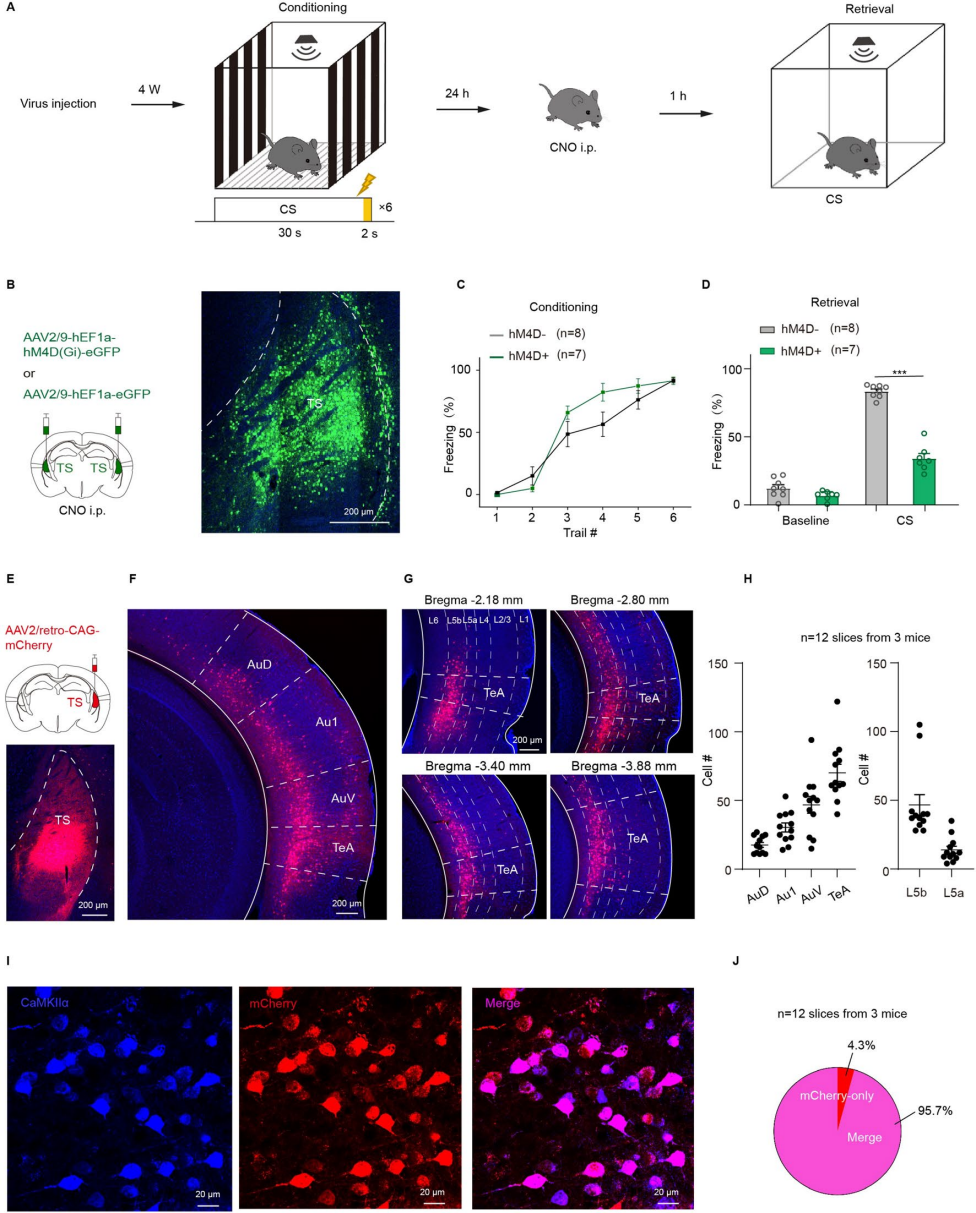
Glutamatergic neurons in the TeA project to the TS. (**A**) Protocol for retrieval testing. CNO i.p. (**B**-**D**) The injection sites and expression of hM4D in TS with CNO i.p., and the freezing% during fear conditioning (**C**) and retrieval testing (**D**). hM4D- vs. hM4D+, *t*_(16.16)_ = 12.79, *p* = 7.19 × 10^−10^ (**D**), *t* test for CS. (**E**) Injection of AAV2/retro-CAG-mCherry in TS (top) and expression(bottom). (**F**) Retrogradely labeled neurons projecting to TS in AuD, Au1, AuV and TeA. (**G**) Retrogradely labeled the TeA_TS_ neurons from rostral to caudal, located as the range from the bregma. (**H**) Distribution statistics. (**I**) Immunohistochemical staining of TeA. Blue: CaMKIIα; red: retrogradely labeled the TeA_TS_ neurons; purple: merge neurons. (**J**) Percentage of CaMKIIα-positive (merge) cell number in retrogradely labeled neurons (red).

TS is not only a critical nucleus involved in defensive responses^36^ but has also been found to play a pivotal role in auditory fear learning and memory^23–25^. To determine the function of the TS in the process of auditory fear retrieval to pure tones, a chemogenetic approach was employed to manipulate neurons in TS using Clozapine N-oxide (CNO) (Fig. 1A), and variations in pure-tone fear retrieval were assessed. Gi-coupled hM4D (hM4D(Gi)) was expressed in neurons of bilateral TS (Fig. 1B) by injection of the virus (AAV2/9-hEF1a-hM4D(Gi)-eGFP or AAV2/9-hEF1a-eGFP as the control). After the animals were successfully trained with pure tone fear conditioning (Fig. 1C), CNO was delivered through intraperitoneal injection (i.p.) to inhibit TS neurons. A 49.29% reduction of freezing was observed during retrieval testing (hM4D-: 83.43 ± 1.65%; hM4D+: 34.14 ± 3.62%; hM4D- vs. hM4D+, *t*_(16.16)_ = 12.79, *p* = 7.19 × 10^−10^, *t* test for CS; Fig. 1D). These findings indicate that freezing behavior for pure tone fear retrieval is due to the activation of TS neurons.

In addition, TS receives projections from multiple cortices^11,18,20–22,37,38^ including TeA^11,18,24^. To verify these projections, the characteristics of cells projecting to TS were investigated by injecting a retrograde labeling virus (AAV2/retro-CAG-mCherry) into the TS (Fig. 1E). Red labeled cell bodies were observed in TeA (Fig. 1F), revealing a neural circuit of the TeA-TS. The labeled neurons in TeA were predominantly located from rostral to caudal (Fig. 1G). In particular, cortical neurons projecting to TS display a progressive increase in distribution from the dorsal auditory cortex (AuD), primary auditory cortex (Au1), ventral auditory cortex (AuV) to TeA (Fig. 1H, left) and convergence to L5b (Fig. 1H, right). To confirm the type of TeA neurons projecting to TS (TeA_TS_), brain slices were stained for calcium/calmodulin-dependent protein kinase IIα (CaMKIIα) protein, a selective marker for excitatory glutamatergic neurons. The results highlighted that the TeA_TS_ cells were mainly projection neurons expressing CaMKIIα (Merge/mCherry = 95.7%) (Fig. 1I and J). Therefore, a neural circuit connecting TeA and TS has been identified, which is hypothesized to be involved in auditory fear retrieval.

### TeA to TS circuit is involved in pure tone fear behavior

**Fig. 2:**
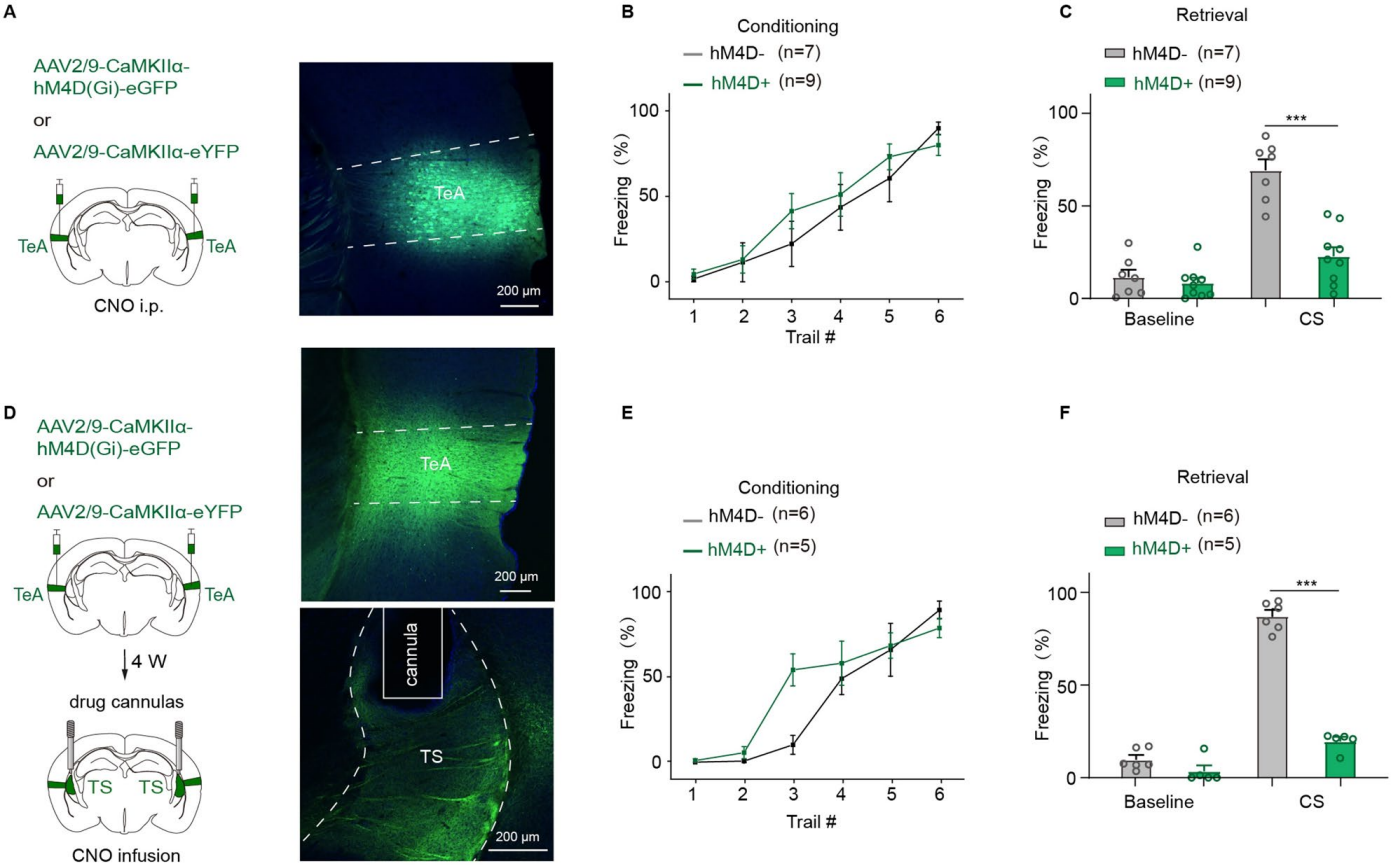
Chemogenetic inhibition of TeA-TS neural circuit impaired pure tone auditory fear retrieval. (**A**) The injection sites and expression of hM4D in TeA with CNO i.p., and the freezing% during fear conditioning (**B**) and retrieval testing (**C**). hM4D- vs. hM4D+: *t*_(14)_ = 5.97, *p* = 3.4 × 10^−5^ (**C**); *t*_(9)_ = 16.69, *p* = 4.44 × 10^−8^ (**F**), *t* test for CS. (**D**) The injection sites and expression of hM4D in TeA with CNO infused in TS locally, and the freezing% during fear conditioning (**E**) and retrieval testing (**F**).

Both TeA^11^ and TS^23–25^ are involved in the learning and memory of pure tone auditory fear conditioning. It is necessary to know whether TeA works through the projection to TS. To address this question, a chemogenetic approach was employed to manipulate either TeA neurons or TeA-TS projections using CNO, as described previously (Fig. 1A). Gi-coupled hM4D (hM4D(Gi)) was expressed in neurons of bilateral TeA (Fig. 2A) by injection of the virus (AAV2/9-CaMKIIα-hM4D(Gi)-eGFP or AAV2/9-CaMKIIα-eYFP as the control). After the animals were successfully trained with pure tone fear conditioning (Fig. 2B), CNO was delivered through intraperitoneal injection (i.p.) to inhibit TeA neurons. A 46.46% reduction of freezing was observed during retrieval testing (hM4D-: 69.12 ± 6.04%; hM4D+: 22.66 ± 5.01%; hM4D- vs. hM4D+, *t*_(14)_ = 5.97, *p* = 3.4 × 10^−5^, *t* test for CS; Fig. 2C). Next, the effect of TeA-TS circuit on fear retrieval was examined using the same approach. To silence the TeA to TS circuit, CNO was locally infused in TS of the mice expressing hM4D(Gi) in the TeA using cannulas (Fig. 2D). This manipulation also impaired fear behavior expression (Fig. 2F). 

These studies demonstrate that the activation of the TeA-TS neural circuit is essential for the pure tone auditory fear retrieval.

### Calcium signaling in TeA_TS_ neurons is significantly enhanced during fear retrieval

**Fig. 3:**
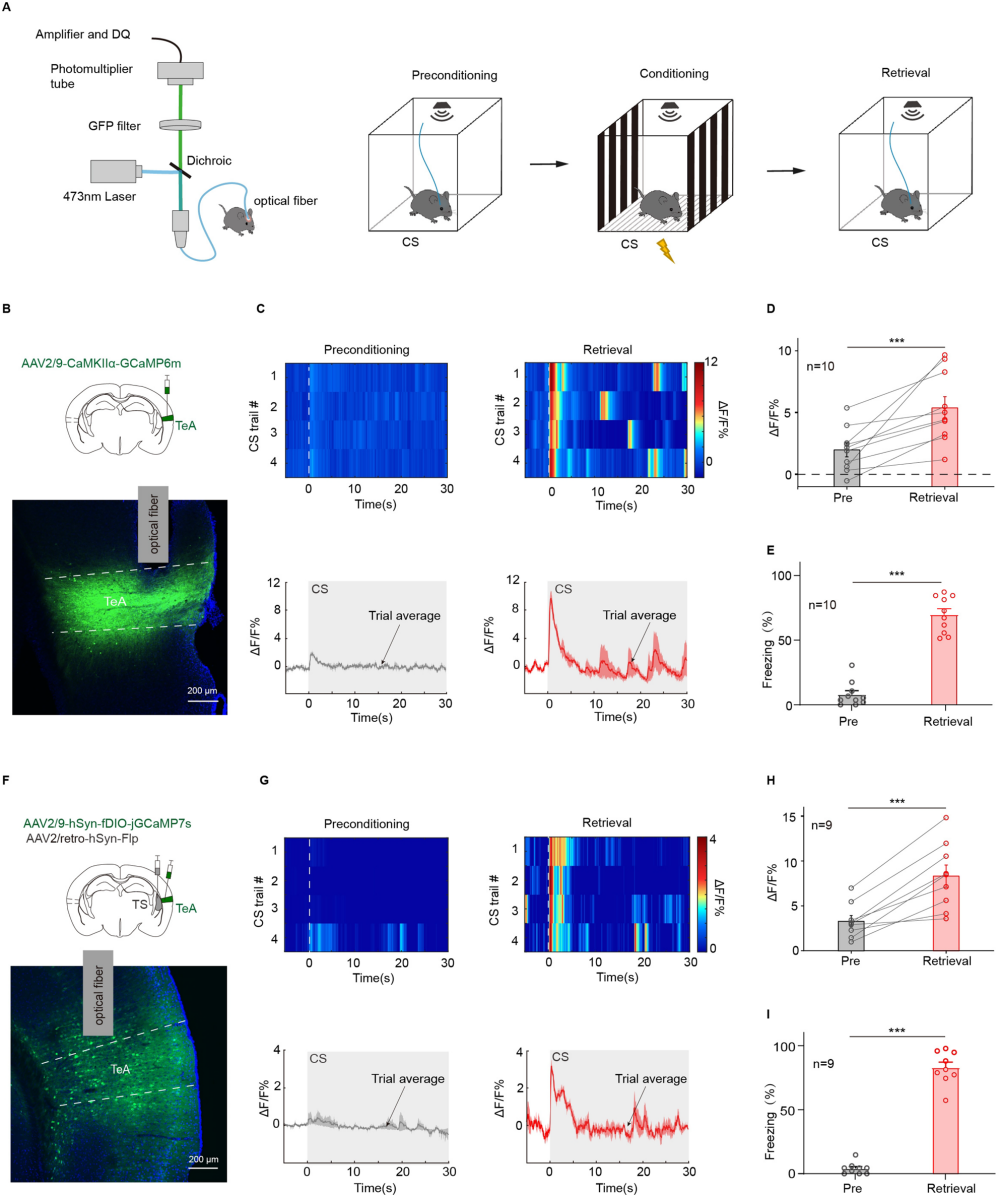
Calcium signal enhancement of TeA_TS_ neurons during the fear retrieval testing. (**A**) Schematic of fiber photometry system and the protocol. Preconditioning and retrieval CS testing in a context distinct from the training arena. (**B**) Top, injection of AAV2/9-CaMKIIα-GCaMP6 m in unilateral TeA. Bottom, GCaMP6 m (green) expression at the injection site. The gray column represents the optical fiber. (**C**) Heat maps (top) and mean Ca^2+^ traces ± s.e.m. (shaded area) (bottom) for a representative animal. (**D**-**E**) ΔF/F% (**D**) and CS-evoked freezing (**E**) during the retrieval test for animals during preconditioning (gray) and retrieval (red) in (**B**). ΔF/F%: preconditioning vs. retrieval, *t*_(9)_ =−4.16, *p* = 0.0025, two-sided paired *t* test (**D**). Freezing%: preconditioning vs. retrieval, *t*_(9)_ =−12.24, *p* = 0.1 × 10^−5^, two-sided paired *t* test (**E**). (**F**) Top, injection of AAV2/9-hSyn-fDIO-jGCaMP7s in unilateral TeA and injection of AAV2/retro-hSyn-Flp in ipsilateral TS. Bottom, jGCaMP7 s (green) expression at TeA. The gray column represents the optical fiber. (**G**) Heat maps (top) and mean Ca^2+^ traces ± s.e.m. (shaded area) (bottom) for a representative animal. (**H-I**) ΔF/F% (**H**) and CS-evoked freezing% (**I**) during the retrieval test for animals during preconditioning (gray) and retrieval (red) in (**F**). ΔF/F%: *t*_(8)_=−5.96, *p* = 3.37 × 10^−4^, two-sided paired *t* test(**H**). Freezing%: preconditioning vs. retrieval, *t*_(8)_=−19.94, *p* = 4.18 × 10^−8^, two-sided paired *t* test(**I**).

TeA_TS_ is involved in behavioral expression of pure tone auditory fear retrieval (Fig. 2D and F). In order to better show the changes in the response of TeA_TS_ neurons to the conditioned sound during fear retrieval, fiber photometry was utilized to monitor real-time changes in calcium signals within specific brain regions^39^ and neuronal types in mice before and after training (Fig. 3A). Excitatory neurons in TeA were infected with AAV2/9-CaMKIIα-GCaMP6 m (Fig. 3B). After successful training, CS-related calcium signal in retrieval was much reinforced compared to that in preconditioning test (Fig. 3C). Statistical analysis indicated a 5.02% difference between before and after training (ΔF/F%: preconditioning: 3.29 ± 0.63%; retrieval: 8.31 ± 1.23%, preconditioning vs. retrieval, *t*_(9)_=−4.16, *p* = 0.0025, two-sided paired *t* test; Fig. 3D), which mirrored freezing behavior changes (Freezing%: preconditioning: 7.97 ± 3.02%; retrieval: 69.83 ± 4.47%; preconditioning vs. retrieval, *t*_(9)_ =−12.24, *p* = 1.0 × 10^−4^, two-sided paired *t* test; Fig. 3E). Next, jGCaMP7s were specially expressed in TeA_TS_ through injection of AAV2/9-hSyn-fDIO-jGCaMP7s in TeA and AAV2/retro-hSyn-Flp in TS (Fig. 3F). Similar results showing higher calcium signal in retrieval were observed, which was supposed to come from TeA neurons directly projected to TS (Fig. 3G and I). These results clearly indicate that calcium signaling in TeA_TS_ neurons is significantly enhanced following auditory fear conditioning.

### Distribution of inhibitory neurons in TeA

**Fig. 4:**
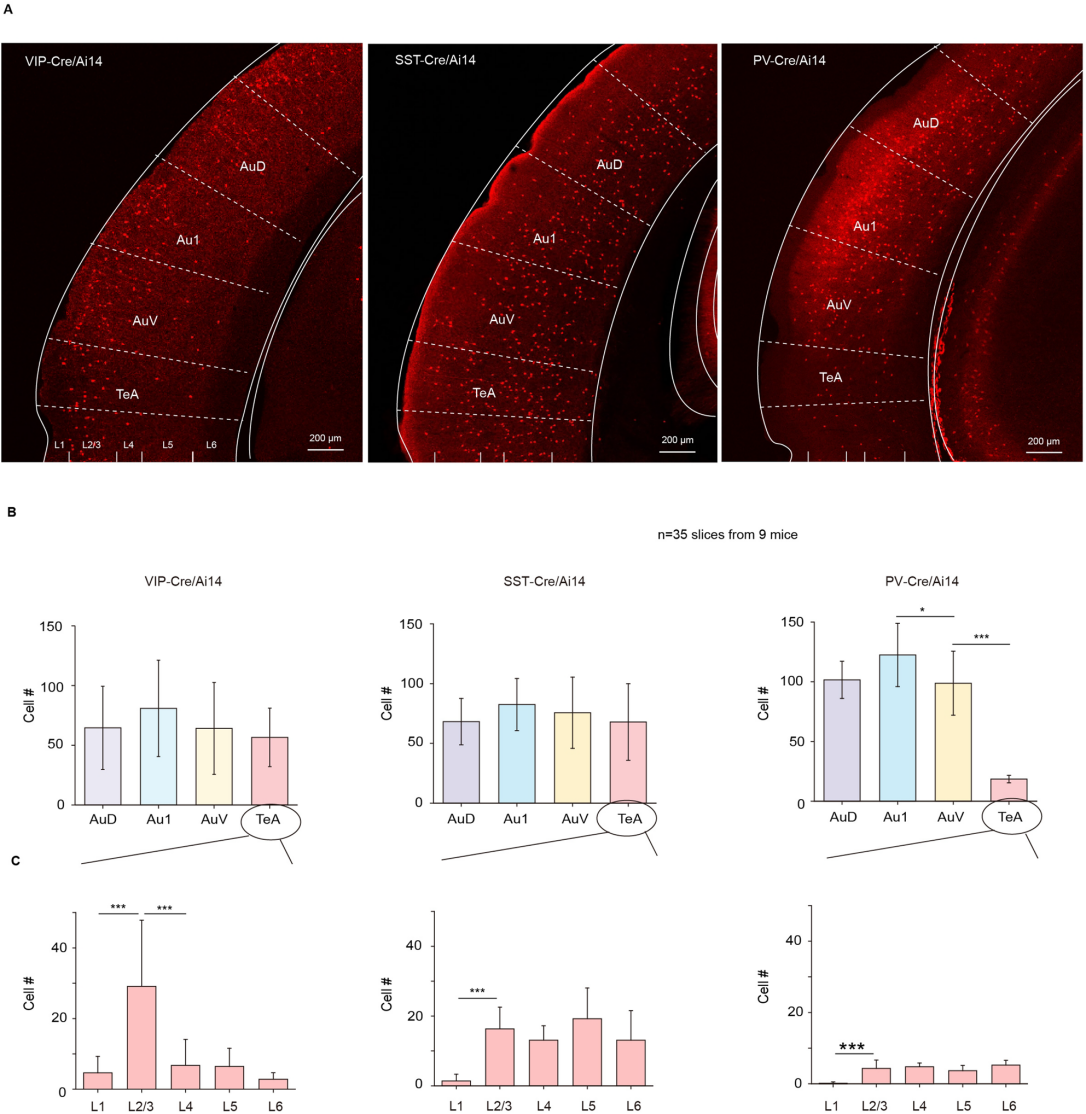
Distribution of three major types of inhibitory neurons in the TeA. (**A**) Examples of the distribution of VIP or SST or PV in AuD, Au1, AuV and TeA. (**B**) Distribution statistics of VIP, SST, and PV in ipsilateral AuD, Au1, AuV, TeA. VIP: *F*_(3,28)_ = 0.68, *p* = 0.572. SST: *F*_(3,36)_ = 0.7, *p* = 0.5593. PV: *F*_(3,48)_ = 65.21, *p* = 1.0 × 10^−13^, One way ANOVA with Tukey’s multiple comparisons test. (**C**) Distribution statistics of VIP, SST, and PV in different layers of the TeA. VIP: *F*_(4,50)_ = 13.14, *p* = 2.19 × 10^−7^. SST: *F*_(4,55)_ = 13.21, *p* = 1.31 × 10^−7^. PV: *F*_(4,45)_ = 21.6, *p* = 3.35 × 10^−11^. One-way ANOVA with Tukey’s multiple comparisons test.

Numerous experiments have demonstrated that inhibitory neurons, such as vasoactive intestinal peptide (VIP), somatostatin (SST) and parvalbumin (PV) neurons, play key roles in fear learning and memory^34,35,40^. To determine the distribution of three major inhibitory neuron types in TeA and AC, VIP-Cre, SST-Cre, and PV-Cre mice were crossed with Ai14 mice, and interneuron distribution was examined across AuD, Au1, AuV, and TeA (Fig. 4A). Statistical analysis revealed that SST and VIP were widely distributed in these cortices, whereas PV neurons were more prevalent in AC but less in TeA (Fig. 4B). Further analysis indicated that SST neurons were located across L2-6 of TeA, whereas VIP neurons were notably concentrated in L2/3 (Fig. 4C).

### Activation of SST neurons impairs pure tone auditory fear retrieval

**Fig. 5:**
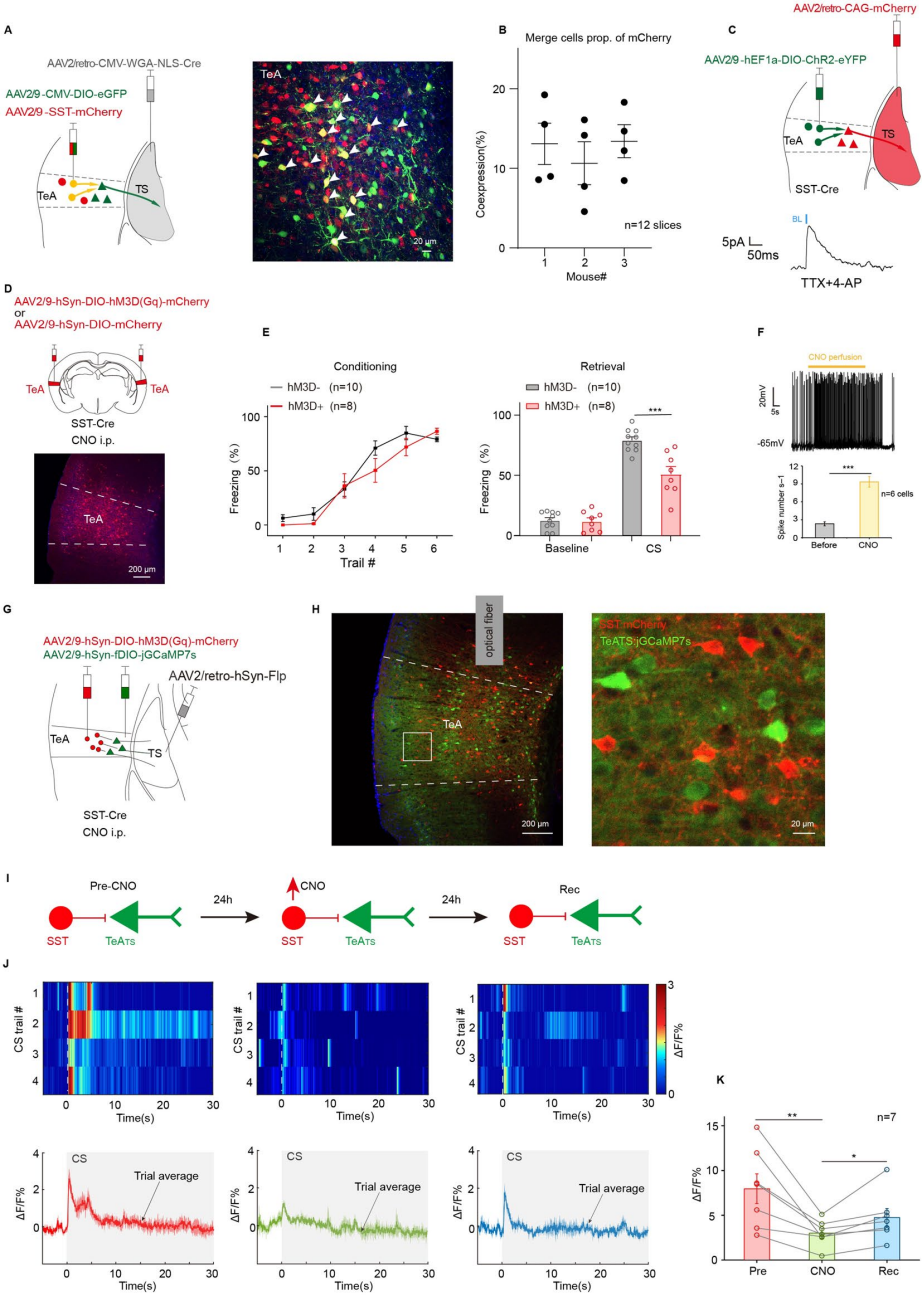
Activation of SST neurons significantly reduces calcium signaling in TeA_TS_ neurons during auditory fear detection. (**A**) Left, injection of AAV2/retro-CMV-WGA-NLS-Cre in TS and a mixture of AAV2/9-CMV-DIO-eGFP and AAV2/9-SST-mCherry in TeA. Right: neurons co-labeled mCherry (red) and eGFP (green) that are SST neurons projecting to the TeA_TS_. (**B**) Proportion of merge neurons to SST (red). (**C**) Top, injection of AAV2/9-hEF1a-DIO-ChR2-eYFP in TeA of SST-Cre mice, and AAV2/retro-CAG-mCherry in TS. In *vitro* recording of TeA_TS_ neurons under blue light. Bottom,  IPSC during perfusion with TTX and 4-AP. (**D**) Top, injection of AAV2/9-hSyn-DIO-hM3D(Gq)-mCherry or AAV2/9-hSyn-DIO-mCherry in TeA. Bottom, hM3D(red) expression in TeA. (**E**) Freezing% during fear conditioning (left) and retrieval testing (right) in mice of (**D**). CS: hM3D- vs. hM3D+, *t*_(16)_ = 4.28, *p* = 5.71 × 10^−4^, *t* test. (**F**) Top, raw trace of current-clamp recording from an hM3D(Gq)-expressing TeA neuron in the slice preparation. Bottom, average spontaneous spike frequencies before and after perfusion with CNO, *F*_(1,10)_ = 55.13, *p* = 2.3 × 10^−5^, One-way ANOVA with Bonferroni test. (**G**) Injection of AAV2/retro-hSyn-Flp in TS and a mixture of AAV2/9-hSyn-DIO-hM3D(Gq)-mCherry and AAV2/9-hSyn-fDIO-jGCamp7s in TeA of SST-Cre mice. (**H**) Left, virus expression in TeA. Right, expression of SST neurons (red) and TeA_TS_ neurons (green) in TeA. (**I**) Procedure schematic of fiber photometry during retrieval testing for three days: pre-CNO, CNO, rec: recovery from CNO. (**J**) Heat maps (top) and mean Ca^2+^ traces ± s.e.m. (shaded area) (bottom) of calcium signaling from TeA_TS_ neurons. (**K**) Statistic of TeA_TS_ neurons activity during retrieval test. *F*_(3,18)_ = 4.712, *p* = 0.0134, One-way repeated-measures ANOVA.

Because inhibitory neurons that directly regulate glutamatergic neurons are typically SST or PV neurons^27^ and given that TeA contains only a small population of PV neurons, the study primarily focused on SST neurons. To test this, two viruses (AAV2/9-CMV-DIO-eGFP and AAV2/9-SST-mCherry) were injected into TeA, and a retrograde virus (AAV2/retro-CMV-WGA-NLS-Cre) was injected into TS (Fig. 5A, left). The WGA secrets a protein signaling peptide, connects to the Cre, and allows the virus to express retrogradely and transsynaptically. Then, it is combined with the corresponding DIO virus. The expression of eGFP would be in TeA_TS_ and its upstream mCherry-expressing SST neurons, which were co-labeled and showed in yellow. Different neurons labeled in green or, red, and yellow were indeed observed (Fig. 5A, right). The co-labeled cells (yellow), assumingly SST neurons in TeA innervating TeA_TS_, composed of 15.5 ± 4.2% of the total SST neurons (yellow to red ratio, Fig. 5B). Next, in vitro electrophysiological studies on brain slices using the patch clamp technique were performed to verify the inhibitory input of SST neurons onto TeA_TS_ neurons. Virus (AAV2/9-hEF1a-DIO-ChR2-eYFP) was injected into TeA, and a retrograde virus (AAV2/retro-CAG-mCherry) was injected into TS of SST-Cre mice (Fig. 5C, top). When TTX (a sodium channel blocker, 1 µM) and 4-AP (a potassium channel blocker, 1 mM) were perfused, the IPSCs can be recorded (Fig. 5C, bottom). This result proves that SST has direct inhibitory input on TeA_TS_.

Based on the distribution of VIP, SST, PV interneurons in TeA, TeA_TS_ converge to L5b (Fig. 1H, right), which matches the distribution of SST neurons. Therefore, SST neurons were hypothesized to be involved in pure tone fear retrieval. To test this, bilateral TeA of SST-Cre mice were injected with chemogenetic virus (AAV2/9-hSyn-DIO-hM3D(Gq)-mCherry) expressing Gq-coupled hM3D (hM3D(Gq)) (Fig. 5D). Twenty-four hours after conditioning, the successfully trained mice (Fig. 5E, left) were given CNO (i.p.) to excite SST neurons expressing hM3D(Gq). As expected, these mice exhibited a 27.34% reduction in CS-related freezing behavior (hM3D-: 81.59 ± 2.83%; hM3D+: 54.25 ± 4.62%; hM3D- vs. hM3D+, *t*_(16)_ = 4.28, *p* = 0.0057, *t* test for CS; Fig. 5E, right). The influence of CNO on the activity of SST neurons expressing hM3D(Gq) was also validated in vitro brain slices using patch-clamp techniques (Fig. 5F, top). An increase in spike rate of the SST neurons following CNO perfusion delivery was observed (Fig. 5F, bottom).

Furthermore, to investigate whether the enhancement of TeA_TS_ is gated by the SST neurons, a Flp virus system was injected into the TS of SST-Cre mice to express jGCaMp7s in the TeA_TS_ neurons, while the DIO virus was used to express hM3D(Gq) in the SST neurons (Fig. 5G and H). After the infection, the calcium signal of the TeA_TS_ excitatory neurons can be monitored, and the SST neurons can be activated with CNO. The first retrieval test was conducted after training. One day after the first retrieval test, a secondary retrieval test was performed following CNO (i.p.) administration. A third retrieval test was performed one day after the CNO injection to verify the recovery from the impact of the SST activation (Fig. 5I). Compared with the first retrieval testing result, there was reduction in calcium signals during secondary retrieval testing when the SST neurons were activated with CNO. The calcium signals tended to return to the initial testing levels at the third retrieval testing (Fig. 5J). Data processing of calcium signal detection over three consecutive days showed a significant change in ΔF/F% during CNO treatment, which seemed to be recovering one day after conditioning (Fig. 5K). Simultaneously, calcium signals in SST neurons were recorded both pre- and post-fear conditioning. A subset of mice exhibited reductions in SST neuronal calcium signal following fear conditioning (Supplementary Fig. 2). These findings demonstrate that the enhancement of TeA_TS_, as pure tone auditory fear retrieval, is gated by SST neurons.

### Inhibition of VIP neurons impairs pure tone auditory fear retrieval

**Fig. 6:**
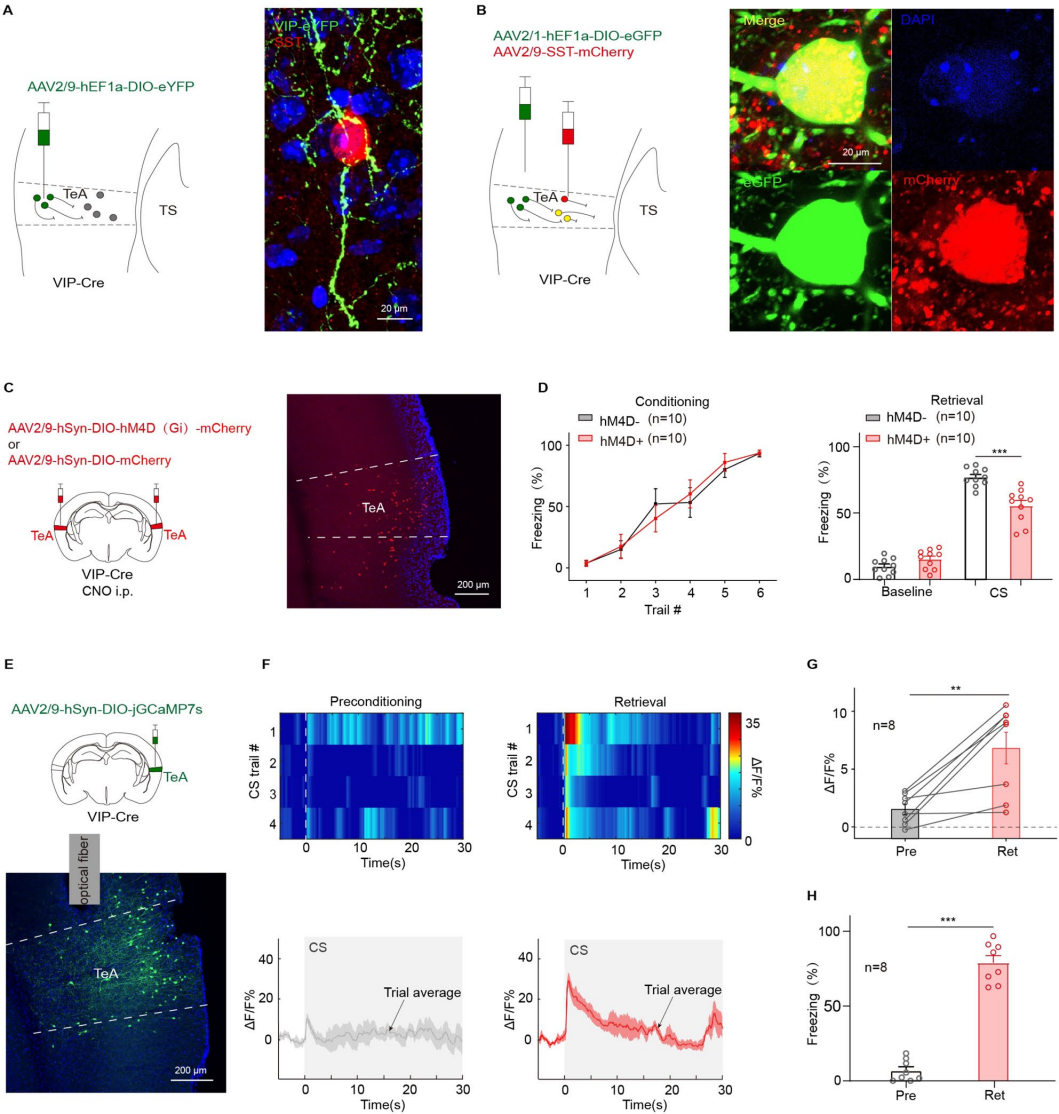
Inhibition of VIP neurons in TeA effectively impaired pure tone auditory fear retrieval. (**A**) Left, injection of AAV2/9-hEF1a-DIO-eYFP in unilateral TeA of VIP-Cre mice. Right: example images of a VIP neuron’s axon (green) and SST neuron’s body (red) within immunofluorescence staining. (**B**) Left, schematic of VIP-Cre mice injected with AAV2/1-hEF1a-DIO-eGFP and AAV2/9-SST-mCherry virus in TeA. Right, an SST neuron(yellow) receiving projections from an upstream VIP neuron. (**C**) Left, injection of AAV2/9-hSyn-DIO-hM4D(Gi)-mCherry or AAV2/9-hSyn-DIO-mCherry in bilateral TeA. Right, hM4D(Gi) (red) expression in TeA. (**D**) Freezing% during fear conditioning (left) and retrieval test (right) in mice of (**C**). CS: hM4D- vs. hM4D+, *t*_(18)_ = 4.71, *p* = 1.74 × 10^−5^, *t* test. (**E**) Top, injection of AAV2/9-hSyn-DIO-jGCaMP7s in unilateral TeA of VIP-Cre mice. Bottom, jGCaMP7s (green) expression at the injection site. (**F**) Heat maps (top) and mean Ca^2+^ traces ± s.e.m. (shaded area) (bottom) of calcium signaling from VIP neurons during preconditioning and retrieval test. (**G**-**H**) ΔF/F%(G) and CS-evoked freezing% (**H**) during the retrieval test for animals during preconditioning (gray) and retrieval (red) in (**E**). ΔF/F%: preconditioning vs. retrieval, *t*_(7)_=−4.19, *p* = 0.0041, two-sided paired *t* test; Freezing%: preconditioning vs. retrieval, *t*_(7)_=−15.534, *p* = 0.1 × 10^−5^, two-sided paired *t* test.

VIP neurons often exhibit a targeted projection relationship with SST neurons^26,40^. Therefore, it was postulated that VIP neurons mediate SST neuron suppression. To test this hypothesis, the virus (AAV2/9-hEF1a-DIO-eYFP) was injected into TeA of VIP-Cre mice (Fig. 6A, left). Six weeks later, the VIP neurons and their fibers expressed eYFP in green. The immunohistochemical staining of the frozen brain slices with the antibodies against SST showed SST neurons in red (Fig. 6A, right). Morphological synaptic contacts (the points in yellow) were observed between VIP and SST neurons. To enhance visualization of morphological synaptic contacts, cell bodies across multiple levels were highlighted using a transsynaptic virus (AAV2/1-hEF1a-DIO-eGFP). Combined with another virus (AAV2/9-SST-mCherry) at a 1:1 ratio, it was injected into the TeA of VIP-Cre mice (Fig. 6B, left). This approach enabled clear visualization of the cell bodies of VIP neurons (green), SST neurons (red) and SST neurons innervated by VIP neurons (yellow) (Fig. 6B, right). These results suggest that the VIP neurons innervate the downstream SST neurons forming a VIP-SST disinhibitory circuit in TeA.

hM4D(Gi) was further expressed in TeA VIP neurons of VIP-Cre mice (Fig. 6C). The day after the mice were successfully trained (Fig. 6D, left), the effect of chemogenetic suppression with CNO on retrieval was tested. Statistical analysis revealed that these mice exhibited a 21.32% decrease in freezing following CNO (i.p.) administration (hM4D-: 76.95 ± 2.15%; hM4D+: 55.43 ± 4.03%; hM4D- vs. hM4D+, *t*_(18)_ = 4.71, *p* = 1.74 × 10^−5^, *t* test for CS; Fig. 6D, right). These results indicate that VIP neurons have a crucial role in pure tone auditory fear retrieval.

TeA has fewer PV neurons but an abundance of VIP and SST neurons (Fig. 4A and B). Therefore, it is postulated that the suppression of SST neurons originates from VIP neurons. To explore this, jGCaMP7s were expressed in VIP neurons of VIP-Cre mice (Fig. 6E). The calcium signal of VIP neurons in response to CS was notably potentiated, showing striking differences between preconditioning and retrieval in both calcium signal (ΔF/F%: preconditioning: 1.54 ± 0.45%; retrieval: 6.82 ± 1.36%, preconditioning vs. retrieval, *t*_(7)_=−4.19, *p* = 0.0041, two-sided paired t test; Fig. 6G) and freezing behavior (Freezing%: preconditioning: 6.81 ± 2.55%; retrieval: 78.92 ± 4.74%; preconditioning vs. retrieval, *t*_(7)_=−15.53, *p* = 1.0 × 10^−4^, two-sided paired t test; Fig. 6H). These results confirm that the VIP neurons in TeA are activated in the long-term and contribute to the pure tone auditory fear retrieval.

### Pure tone fear conditioning reinforces tea cholinergic release

**Fig. 7:**
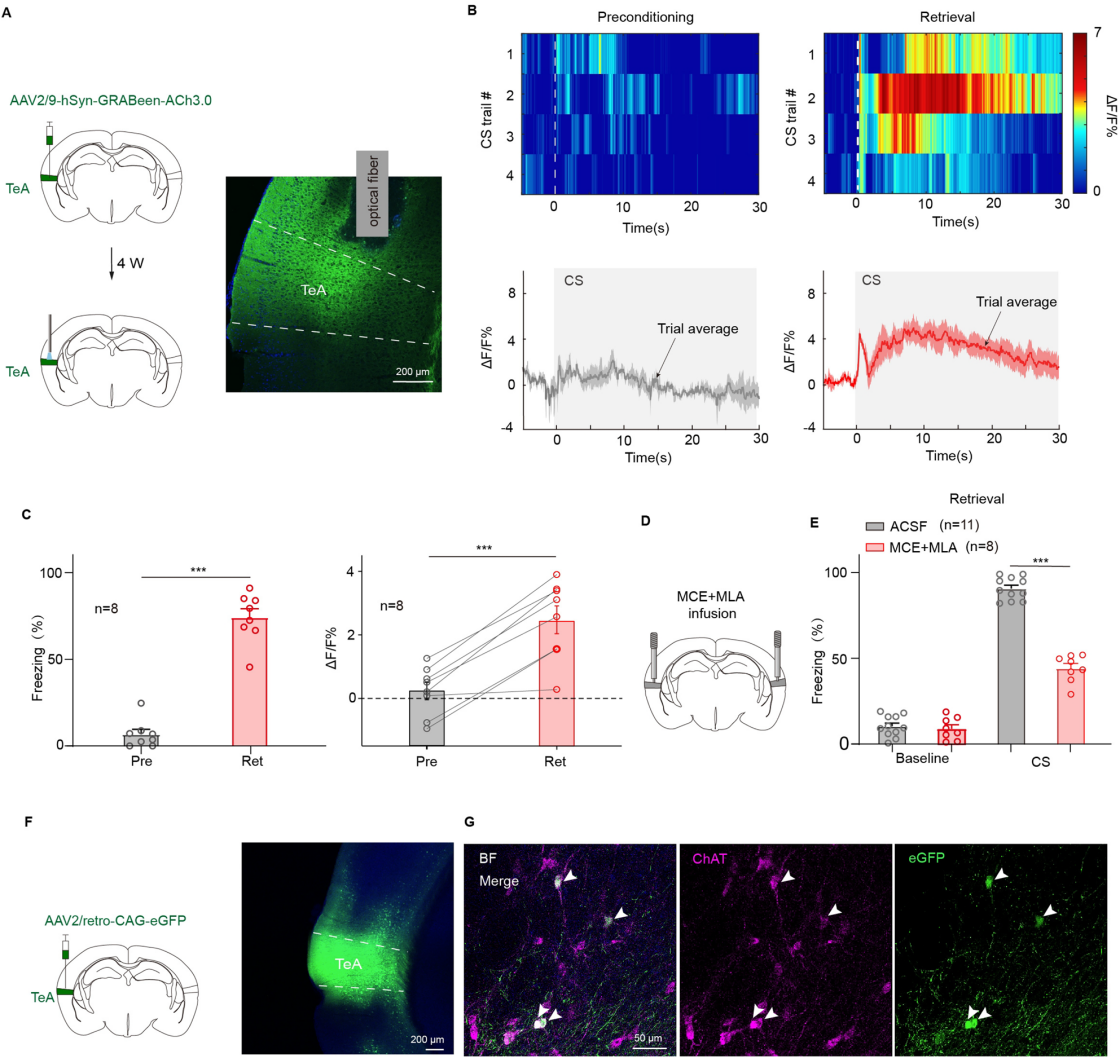
Enhancement of ACh signaling in the TeA during auditory fear testing. (**A**) Injection and fiber photometry site (left) and expression (right) of AAV2/9-hSyn-GRABeen-ACh3.0 in unilateral TeA. (**B**) Heat maps (top) and mean Ach traces ± s.e.m. (shaded area) (bottom) of Ach signaling from TeA during preconditioning and retrieval test. (**C**) Statistics of CS-evoked freezing% (left) and ΔF/F% (right). Preconditioning vs. retrieval, *t*_(7)_=−6.89, *p* = 2.33 × 10^−4^, two-sided paired *t* test(left); *t*_(7)_=−10.54, *p* = 1.5 × 10^−5^, two-sided paired *t* test(right). (**D**) Injection of MCE and MLA into bilateral TeA before retrieval test. (**E**) Freezing during retrieval testing in mice of (**D**). CS: ACSF vs. MCE + MLA, *t*_(17)_ = 13.97, *p* = 9.6 × 10^−11^, *t* test. (**F**) Injection (left) and expression (right) of AAV2/retro-CAG-eGFP in unilateral TeA. (**G**) An example showing TeA receiving projections from acetycholinergic neurons expressing ChAT (red) in BF.

Subsequent investigation focused on TeA afferent inputs. Given established evidence that AC modulation by BF cholinergic signaling enhances auditory fear conditioning in mice^35^. We next examined whether TeA similarly receives cholinergic projections that trigger auditory fear retrieval. To explore this, first, a virus encoding both GRABeen and ACh3.0 sensor was injected into TeA for in vivo fiber photometry (Fig. 7A). Following fear conditioning, the ACh signal elicited by pure tone significantly increased, and more importantly, maintained a high-level signal throughout the sound presentation period (Fig. 7B). Statistical analysis indicated that the CS-related ACh release was significantly more abundant for the postconditioning (retrieval) compared to the preconditioning, which was in accord with the change of freezing behavior (Freezing%: preconditioning: 6.74 ± 2.83%; retrieval: 74.1 ± 5.06%; preconditioning vs. retrieval, *t*_(7)_=−10.54, *p* = 1.5 × 10^−5^, two-sided paired t test; Fig. 7C left; ΔF/F%: preconditioning: 0.23 ± 0.27%; retrieval: 2.47 ± 0.44%, preconditioning vs. retrieval, *t*_(7)_= −6.89, *p* = 2.33 × 10^−4^, two-sided paired *t* test; Fig. 7C right).

Conversely, two nicotinic acetylcholine receptor (nAChR) antagonists, mecamylamine (MCE) and methyllycaconitine (MLA), were locally applied to TeA (Fig. 7D) before retrieval testing, 0.1 µl per hemisphere respectively. Compared to the control group, which received artificial cerebrospinal fluid (ACSF), this manipulation strongly resulted in a reduction of fear freezing levels (ACSF: 90.65 ± 1.99% MCE + MLA: 44.14 ± 2.8%; ACSF vs. MCE + MLA, *t*_(17)_ = 13.97, *p* = 9.6 × 10^−11^, *t* test for CS; Fig. 7E). Cortical Ach inputs are considered from BF^41^. Finally, to identify the origin of the ACh transmitter in the TeA, a retrograde labeling virus (AAV2/retro-CAG-eGFP) was injected into the TeA (Fig. 7F), to label the neurons projecting to the TeA. Sequentially, the brain slices expressing eGFP including BF region were immunostained with choline acetyltransferase (ChAT; a marker for cholinergic neurons). The cells expressing eGFP overlapped with ChAT staining (Fig. 7G), confirming that a majority of these eGFP-labeled cells were ChAT-positive neurons. These findings indicate that ACh contributes to pure tone fear retrieval in TeA.

## Discussion

Fear retrieval is crucial for survival and serves as a primary model for understanding how sensory information is linked to innate behaviors through learning. The memory of simple sound (pure tone) threats is believed to reside in TeA^11,12^. However, the microcircuits underlying the function of pure tone fear retrieval and how fear retrieval triggers related behaviors remain poorly understood. In this study, these fundamental questions were addressed using a pure tone auditory fear conditioning model, combined with optogenetic/chemogenetic manipulations, fiber photometry, and electrophysiological recordings. Our experimental results show that inhibition of TS impairs the auditory fear retrieval, including pure tone experiments (Fig. 1B and D) and noise experiments (Supplementary Fig. 1). By injecting retrograde tracers into the TS, it was found that TS receives significantly more input from TeA than from auditory cortex (Fig. 1E and H). Furthermore, immunofluorescence staining revealed that 95.7% of TeA_TS_ neurons are glutamatergic (Fig. 1I and J). Numerous studies have reported that damage to the AC does not affect the learning and memory of pure tone auditory fear conditioning^8–10,42^ whereas damage to the TeA strongly impairs both learning and memory of this conditioning^11^. Thus, the learning and memory of pure tones can occur independently of Au1 but require the presence of the TeA. Moreover, lesions of the TeA in mice lead to long-term deficits in fear memory, highlighting the importance of the TeA in long-term memory storage. Through combined chemogenetics and fiber photometry, the TeA-TS pathway was confirmed to facilitate fear retrieval. Regarding auditory fear conditioning – a form of fear memory mediated by acoustic cues – the process involves two critical phases: (1) sound recognition; (2) followed by memory formation. The TeA exhibits robust sound discrimination capabilities, as evidenced by findings that silencing USV-responsive neurons in TeA impairs auditory-driven maternal preference during pup retrieval assays^16^. Notably, although not all TeA_TS_ neurons are sound-responsive, selective inhibition of acoustically-tuned TeA_TS_ neurons would likely impair auditory fear retrieval. Since the ventrolateral periaqueductal gray (vPAG) is a key brain region for freezing behavior^43^ the TS may mediate this behavior via its projections to the vPAG.

Given the critical role of the TeA in fear retrieval, the connectivity and function of inhibitory neurons were further explored. Sixty years ago, John Z. Young proposed a learning model implicating circuit disinhibition^44^. Subsequent studies have revealed that disinhibition is a common circuit mechanism^30^. Disinhibition is an attractive and powerful learning mechanism because it allows strong activation and concomitant plasticity induction, particularly the VIP-SST disinhibition pattern, which appears to be broadly involved in sensory processing and the regulation of different types of learning^31,45^. In the TeA, SST neurons are innervated and inhibited by VIP neurons (Fig. 6A and B), while VIP neurons are predominantly located in layers 2/3 (Fig. 4A and C), which is not surprising. VIP neurons often serve as disinhibitory gates in the cortex, preferentially driven by long-range excitatory and neuromodulatory projections^45,46^. Interestingly, the cerebral cortex provides input to VIP interneurons through long-range projections, local projections, as well as inputs from the dorsal raphe nucleus and basal forebrain^27,40,47–49^ suggesting that, depending on the nature of the learning task, different projection systems can modulate the microcircuit studied here. Indeed, our results show that the TeA receives cholinergic projections from BF (Fig. 7F and G), and during fear retrieval, the calcium signals of VIP neurons are significantly enhanced in response to the CS (Fig. 6F and G). Given that SST neurons exhibit short-term plasticity^26^ fear retrieval in the TeA-TS pathway may be transmitted through VIP neurons.

An intriguing observation is the scarcity of PV neurons in the TeA (Fig. 3D and E). PV cells are rapidly activated by afferent inputs^50^ and are thought to regulate the output of excitatory neurons with millisecond precision through strong shunting inhibition on cell bodies^51^. IPSPs from PV synapses exhibit rapid suppression at high activity rates^52^ suggesting that PV-mediated inhibition may only be effective over short time scales. This brief window of PV efficacy might be utilized to tightly constrain the temporal precision of the first spike in cortical neurons evoked by sensory inputs, which carries substantial information^26^. Therefore, the lack of PV neurons in the TeA may represent a functional adaptation for receiving more extensive information, where high levels of integration occur. Combined with the TeA’s ability to receive projections from nearly all sensory cortices, this suggests that the TeA is indeed a nucleus closely associated with multisensory integration. Although the roles of VIP-SST, VIP-PV, PV-SST, and GAD-PV in auditory cortex learning and memory processes have been demonstrated^34,35^ the scarcity of PV neurons in the TeA’s cellular composition positions it as a novel model for functional studies.

AC receives cholinergic projections from the BF^53–57^ which enhance fear learning. As a classic neuromodulatory system, the cholinergic system is associated with a range of cognitive functions^58^ from arousal and alertness to attention^19^ and learning and memory^35^. TeA also receives cholinergic projections from the basal forebrain, but its specific functional role remains unclear. Here, the Ach3.0 sensor virus was injected into the TeA of mice to monitor real-time acetylcholine dynamics during auditory fear conditioning (Fig. 7A). Acetylcholine signals were found to be significantly increased during fear retrieval compared to baseline levels (Fig. 7B and C). Subsequently, pharmacological blockade of cholinergic receptors in TeA during retrieval phase was found to impair fear retrieval in mice (Fig. 7D and E). These findings indicate that acetylcholine release in the TeA enhances fear retrieval.

Based on the above experimental results, a disinhibitory microcircuit was identified between VIP and SST neurons in the TeA. Acetylcholine release in the TeA is significantly increased during fear retrieval processes, and the VIP-SST circuit is strongly activated, thereby enhancing fear retrieval in the TeA-TS pathway. This is then translated into behavioral regulation in mice through outputs to TS.

## Methods

### Animals

Experiments were conducted on mice of either sex, aged between postnatal days 42 ~ 60. For the experiments, SST-Cre (stock no. 013044), PV-Cre (stock no. 017320), VIP-Cre (stock no. 010908), Ai14 (stock no. 007914), and C57BL/6 J wild-type mice were utilized. Mice of specific genotypes were acquired from the Jackson Laboratory and maintained on a C57BL/6 J genetic background. Prior to the commencement of experiments, mice were assessed for normal hearing. Mice were maintained at conditions of 22 ± 1 °C temperature and 55 ± 5% humidity, within a 12-hour light/dark cycle, with access to food and water ad libitum. The protocol of the animal study was reviewed and approved by the Animal Care and Use Committee at Southern Medical University, Guangzhou, China. All experiments were conducted with efforts to minimize the use of experimental animals and to reduce their suffering. In our research, intraperitoneal administration of pentobarbital sodium was used to induce anesthesia and euthanasia, with the euthanasia dose being three times the anesthetic dose. All experimental methods were carried out in accordance with relevant guidelines and regulations, and are reported in accordance with the ARRIVE guidelines.

### Viral vectors

For optogenetic experiments, AAV2/9-hEF1a-DIO-ChR2-eYFP (1.89 × 10^13^ vg/ml, S0199, Taitool) was used. For chemogenetics, AAV2/9-CaMKIIα-hM4D(Gi)-eGFP (5.11 × 10^12^ vg/ml, PT-0524, Brainvta), AAV2/9-hEF1a-hM4D(Gi)-eGFP (8.14 × 10^12^ vg/ml, PT-1840, Brainvta), AAV2/9-hSyn-DIO-hM3D(Gq)-mCherry (1.66 × 10^13^ vg/ml S0192, Taitool), AAV2/9-hSyn-DIO-hM4D(Gi)-mCherry (1.11 × 10^13^ vg/ml S0193, Taitool), AAV2/9-CaMKIIα-eYFP (3.48 × 10^12^ vg/ml, PT-0107, Brainvta) and AAV2/9-hEF1a-DIO-eGFP (1.77 × 10^12^ vg/ml, PT-0291, Brainvta) were used. For GCaMP6 m and Flp/Cre-dependent jGCaMP7 s expression, AAV2/9-CaMKIIα-GCaMP6 m (5.25 × 10^12^ vg/ml, PT-0111, Brainvta), AAV2/9-hSyn-fDIO-jGCaMP7s (1.86 × 10^13^ vg/ml, S0861, Taitool), and AAV2/9-hSyn-DIO-jGCaMP7 s (1.77 × 10^13^ vg/ml S0590, Taitool) were used. For ACh expression, AAV2/9-hSyn-GRABeen-ACh3.0 (1.62 × 10^12^ vg/ml S0639, Taitool) was used. To retrogradely and anterogradely express Cre, AAV2/retro-hSyn-Cre (6.28 × 10^12^ vg/ml, PT-0136, Brainvta), AAV2/retro-CMV-WGA-NLS-Cre (3.56 × 10^12^ vg/ml, H17837, OBiO), and AAV2/1-hSyn-Cre (1.00 × 10^13^ vg/ml, PT-0136, Brainvta) were used. To retrogradely express Flp, AAV2/retro-hSyn-Flp (1.37 × 10^13^ vg/ml, S0271, Taitool) was used. For neuronal tracing and cell labelling experiments, AAV2/9-hEF1a-DIO-eYFP (4.36 × 10^12^ vg/ml, AG20296, OBiO), AAV2/retro-CAG-mCherry (5.01 × 10^12^ vg/ml, PT-0105, Brainvta), AAV2/retro-CAG-eGFP (5.70 × 10^12^ vg/ml, PT-0305, Brainvta), AAV2/9-CMV-DIO-eGFP (3.91 × 10^12^ vg/ml, H5010, OBiO), AAV2/9-SST-mCherry (5.57 × 10^12^ vg/ml, PT-1215, Brainvta), and AAV2/1-hEF1a-DIO-eGFP (1.60 × 10^13^ vg/ml, H3303, OBiO) were used.

### Virus injection

All procedures were conducted under aseptic conditions. Mice were anesthetized with pentobarbital sodium (60–70 mg/kg) and placed in a stereotaxic apparatus (RWD, China). Body temperature was maintained at 37 °C using a heating mat. The bregma was designated as the central reference point (0,0,0) for all coordinates. The virus was injected using a glass micropipette (Drummond Scientific, USA) and a microinjection pump (KD Scientific, USA) at 30 nl/min. Stereotaxic coordinates (posterior to bregma, lateral to midline, and below the brain surface in mm) were as follows: TeA: −2.9, 4.7, −1.8; TS: −1.2, 3.2, −2.8. To optimize TeA injection, the animal’ head was rotated 80° horizontally.

Behavioral and electrophysiological experiments commenced 3–4 weeks post-injection for neuronal cell body targeting, or 5–8 weeks for nerve terminal targeting, to ensure full viral expression.

### Fiber/guide cannula implantation

For fiber photometry experiments, optical fiber ferrules (O.D., 200 μm 0.37 numerical aperture (NA), ThinkerTech, Nanjing) were implanted 200 μm above the unilaterally TS or TeA regions.

For the behavioral experiment involving intracranial drug injections, 10 mm long guide cannulas (O.D., 400 μm, I.D., 300 μm, RWD, China) were implanted above the bilateral TeA/TS. The guide cannulas were secured to the skull using dental cement, and stainless steel obturators (O.D., 200 μm, RWD, China) were inserted into the guide cannulas to maintain patency.

All optical fiber ferrules and guide cannulas were implanted to ensure the left and right hemispheres of the mice remained horizontal.

Following the procedure, the animals were removed from the stereotaxic device, revived on a constant-temperature heating mat, and subsequently returned to their cages.

Pre-behavioral experiments animal habilitation preparation.

For all auditory fear conditioning or open field testing, mice underwent required to undergo 3 days of handling to habituate to the experimenter. For fiber photometry, mice were habituated to fiber photometry 3 days in advance; for chemogenetics, mice receiving injections of saline (i.p.) were also habituated 3 days in advance, and mice receiving intracranial injections of drugs or antagonists were acclimatized to sham-injected needle 3 days in advance.

### Sound stimulation

Pure tones were generated using Tucker-Davis Technologies System 3 (TDT 3, Alachua, FL, USA), amplified by an electrostatic speaker driver (ED1), and were delivered through a calibrated free-field speaker (ES1, 12 kHz) positioned 15 cm away from the mouse. Control of the pure tone parameters were managed via BrainWare software on a computer. Before the experiments, the free-field speaker was calibration using microphones (Bruker and Kjaer 4135) alongside an amplifier (Bruker and Kjaer 2610).

In the behavioral experiments, pure tone of specified frequency (12 kHz, 50 ms duration, 5 ms ramp) at 80 dB SPL, while noise (50 ms duration, 5 ms ramp) was also delivered at 80 dB SPL, controlled by a programmable attenuator (PA5), were presented at a rate of 1/s. During fiber photometry, pure tones of specified frequency (12 kHz, 30 s duration, 5 ms ramp) were presented at 80 dB SPL.

### Auditory fear conditioning

In this study, the classical fear conditioning model was used to train mice. Auditory fear conditioning was conducted in a set of specialized instruments (Xinruan, China). All the processes were recorded by a matching video system. During the fear conditioning training, mice underwent individual sessions, and after 120 s, a 30 s CS was delivered, followed by an US (electric foot shock, 0.6 mA, 2 s) during the final 2 s of the CS. All conditioning trainings consist of six trials, with each separated by a 90 s intertrial interval (ITI). Following the final trial, mice were retained in the chambers for an additional 30 s before being returned to their home cages. Auditory fear conditioning was conducted in a specific behavioral context with black and white stripes on all sides.

For the testing phase, mice were introduced into a distinct testing chamber with white interior on all sides. During testing, mice were exposed to the same tones as those used during training. Each test began with a 3-minute habituation period, followed by a 3-minute presentation of pure tones without the US. Between sessions, the conditioning chamber or testing chamber were cleaned with 70% ethanol or 0.2% acetic acid, respectively, and bedding material was refreshed.

The only difference during the fiber optic recording process was that the testing consisted of four trials with a 90 s ITI between each trial.

### Immunohistochemical staining

Mice were perfused with 50 ml of phosphate buffer solution (PBS) followed by 50 ml of 4% paraformaldehyde through the left ventricle. Brain tissues were fixed in 4% paraformaldehyde at 4 ℃ for 24 h, followed by gradient dehydration in 20% and 30% sucrose solutions at 4 ℃. Coronal sections, 50 μm in thickness, were collected using a cryostat (Leica CM1950). The sections were washed three times (5 min each), then subsequently blocked with blocking solution (5% BSA, 0.3% Triton X-100 in PBS) at room temperature for 1 h. Sections were incubated overnight at 4 ℃ with primary antibodies, including rabbit anti-CaMKIIα (1:300; Abcam, ab52476), rabbit anti-SST (1:1000; Peninsula, T-4103), and goat anti-ChAT (1:300; Millipore, AB144P) in antibody diluent (2% BSA, 0.1% Triton X-100 in PBS). Subsequently, the sections were washed three times in PBS (5 min each) and incubated for 2 h at room temperature with specific fluorophore-conjugated secondary antibodies (1:1000; goat anti-rabbit Alexa Fluor 594, Abcam, ab150064), (1:1000; goat anti-rabbit Alexa Fluor 405, Abcam, ab175652) and (1:1000; donkey anti-goat Alexa Fluor 647, Abcam, ab150131) in antibody diluent. After three PBS washes (5 min each), the sections were mounted on glass slides and covered with coverslips.

### Chemogenetics manipulation and drug delivery

For chemogenetics manipulation, CNO (1 mg/kg) was injected(i.p.) 1 h before fear testing. To inhibit specific neural circuits, with the key distinction being the infusion of CNO into the brain 30 min prior to testing. Specifically, a microinjection pump (RWD, China) drew CNO into a 10 µl microinjector (Hamilton), which then delivered the substance into the targeted nuclei at a rate of 200 nl/min via a polyethylene tube (RWD, China) and a 10.2 cm injection needle (O.D., 200 μm, RWD, China) linked to the microinjector, administering a dose of 400 nl on each side. To facilitate drug dispersion, the drug delivery cannula was left in place for 5 min post-infusion before being replaced with a sham cannula.

For the delivery of nAChR antagonists, the solution was prepared as a 1:1 mixture of MLA (0.1 mM, MCE) and MCE (100 mM, MCE).

### Fiber photometry

Calcium and acetylcholine signals were both collected using a fiber photometry system (Thinker Tech, Nanjing). Before each recording experiment, light intensity at the optical fiber’s end was maintained between 40 and 60 µW to minimize photo bleaching. The system operates by emitting a 488 nm wavelength light from a laser (OBIS 488LS, Coherent), which is attenuated by 1% (Daheng Photoelectricity), reflected vertically by a dichroic mirror (MD498, Thorlabs, US), and focused onto the core end of optical fiber through an objective lens (0.3 NA, Olympus, 10x magnification). After passing through the optical fiber (O.D. 200 μm, 0.37 NA, 2 m in length, Newtoon, China) and the dark ceramic ferrule (Newtoon, China), the excitation light stimulates a green fluorescent signal in the target brain area. The excited fluorescence signal is filtered through a band-pass filter (MF525-39, Thorlabs), detected by a photomultiplier tube (PMT, R3896, Hamamatsu), and converted into current and analog voltage signals by an amplifier (C7319, Hamamatsu). These signals are then relayed to the data collection system via a low-pass filter (40 Hz). Digital records with a sampling rate of 100 Hz. After each recording trial, the laser was immediately deactivated to prevent photobleaching. The calcium and acetylcholine signal were then exported to MATLAB for off-line analysis.

The optical fiber on the mouse’s head was connected to the instrument’s optical fiber 10 min prior to testing. Fiber photometry was conducted simultaneously with the behavioral experiment in a dark environment.

### Behavioral quantification

The behavior of the mice was recorded by a camera mounted above the test chamber. The movement speed of the mice was calculated and analyzed by SuperFcs (XinRuan Technologies, China) at 15 frames/s. Instantaneous movement speeds consistently below 2 cm/s or lasting for 1 s were considered “freezing”.

### In vitro electrophysiology

As with our prior experiments, mice were anesthetized using pentobarbital sodium. Following rapid brain dissection, brain tissues were immersed in a cutting solution (comprising 60 mM NaCl, 3 mM KCl 1.25 mM Na_2_HPO_4_, 25 mM NaHCO_3_, 115 mM sucrose, 10 mM glucose, 7 mM MgCl_2_, and 0.5 mM CaCl_2_; pH 7.4, osmolality 300 ~ 305 mOsm/l) to prepare 300 μm thick coronal sections. Sections were transferred to an incubation chamber and submerged in freshly prepared 34 °C ACSF (124 mM NaCl, 2.5 mM KCl, 1.2 mM NaHPO_4_, 25 mM NaHCO_3_, 1 mM MgCl_2_, 2 mM CaCl_2_, and 20 mM glucose; pH 7.4, osmolality 300 ~ 305 mOsm/l) for 30 min. Prior to recording, the incubation chamber was adjusted to room temperature. Both the cutting solution and ACSF were continuously aerated with 95% O_2_ and 5% CO_2_. The sections were then placed in the recording chamber and perfused with ACSF using a perfusion pump (longerpump, China) at a flow rate of 3–4 ml/min.

Neuronal activity was recorded using the whole-cell patch-clamp technique under an upright microscope (Nikon Eclipse FN1, Japan) equipped with DIC optics and filters, visualizing eYFP/eGFP/mCherry with a 40x water immersion objective and a CCD camera (Nikon, Japan). Action potentials were recorded using whole-cell current clamp setups (HEKA EPC800, Digidata1550 DAC) and pCLAMP10.6 software (Axon Instruments). The electrode’s electrolyte solution contained 140 mM gluconate, 9 mM HEPES, 5 mM EGTA, 4 mM Mg-ATP, 0.3 mM GTP, 4.5 mM MgCl_2_, and 4.4 mM sodium creatine phosphate (pH 7.3, 295 mOsm/l). All chemicals were sourced from Sigma-Aldrich.

During the verification of chemogenetic viral efficacy, alterations in the membrane potential of neurons infected by the virus were monitored before and after exposure to a 5 µM CNO bath. In addition, inhibitory postsynaptic currents (IPSCs)were recorded by clamping the cell’s membrane potential at 0 mV. To record light-evoked IPSCs, blue light (5 ms, 1 Hz, 10 mW) was delivered through the objective to the TeA. Light-evoked IPSCs were recorded in neurons superfused with TTX (1 µM) and 4-AP (1 mM) in order to measure synaptic responses.

#### Image and quantification

The sections were imaged using a laser scanning confocal microscope (Nikon A1R, Japan), and the acquired images were analyzed to verify viral expression and staining condition. The remaining sections were photographed under a 10x objective lens, except for those sections requiring observation of the connectivity between neurons, which were photographed using a 60x oil lens. Cell counting was conducted using ImageJ software.

Data processing and Statistical analysis.

For calcium signal recorded data, a custom MATLAB script developed by ThinkerTech facilitates data processing, and the implanted portion of the optical fiber underwent histological examination. Percentage change in signal (ΔF/F%) was calculated by subtracting the peak fluorescence signal during the test (CS or US) period from the peak fluorescence signal during the respective baseline period and dividing the resulting signal by the peak fluorescence signal in the baseline period (ΔF/F%= (FpeakTEST − FpeakBASELINE)/FpeakBASELINE).

All data underwent statistical analysis using SPSS (Version 20, IBM). The Shapiro-Wilk test was first applied to examine whether samples had a normal distribution. In the case of a normal distribution, one-way repeated-measures ANOVA or two-sided paired t test was applied. OriginPro 2022 (OriginLab Corporation) or GraphPad Prism 9.2 (GraphPad Software) were used for statistical analysis and graphing. Data were presented as mean ± s.e.m. Unless otherwise specified. In this study, all the representative cases are followed by a population summary to demonstrate the repeatability. No statistical methods were used to pre-determine sample sizes but our sample sizes are similar to those reported in previous publications. Significance levels are indicated as *P < 0.05, **P < 0.01, and ***P < 0.001. NS indicates not significant.

## Electronic supplementary material

Below is the link to the electronic supplementary material.

Supplementary Material 1

## Data Availability

The datasets used and/or analysed during the current study available from the corresponding author on reasonable request.
