# Supplementary material for: A disinhibitory microcircuit in the temporal association cortex for fear retrieval to pure tones
Source: Sci Rep. 2025 Jul 1;15:20457. doi: 10.1038/s41598-025-05566-0 (PMC12216945; doi:10.1038/s41598-025-05566-0)
Supplement: Supplementary file 1 — Supplementary Material 1 [file 41598_2025_5566_MOESM1_ESM.pdf]

A disinhibitory microcircuit in the temporal association cortex for fear retrieval to pure tones

Rui Cheng[1], Wen Zhong[2**], Yangqiu Yan[1], Linhui Yao[1], Peiran Yin[2], Ziyi Xu[1], Xiaoxia Qin[3], Jie Tan[2], Yingying Zeng[2], Jinhua Liu[3], Zhongju Xiao[1, 3*]

Supplementary figures:

Supplementary Fig.1

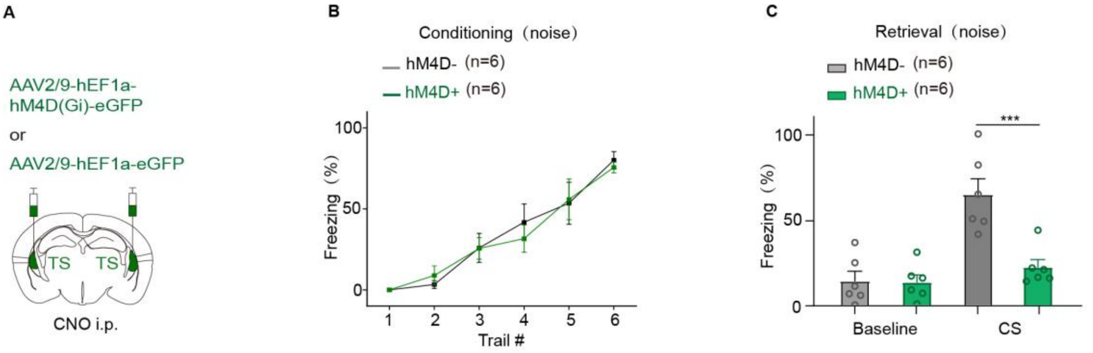

Supplementary Fig.2

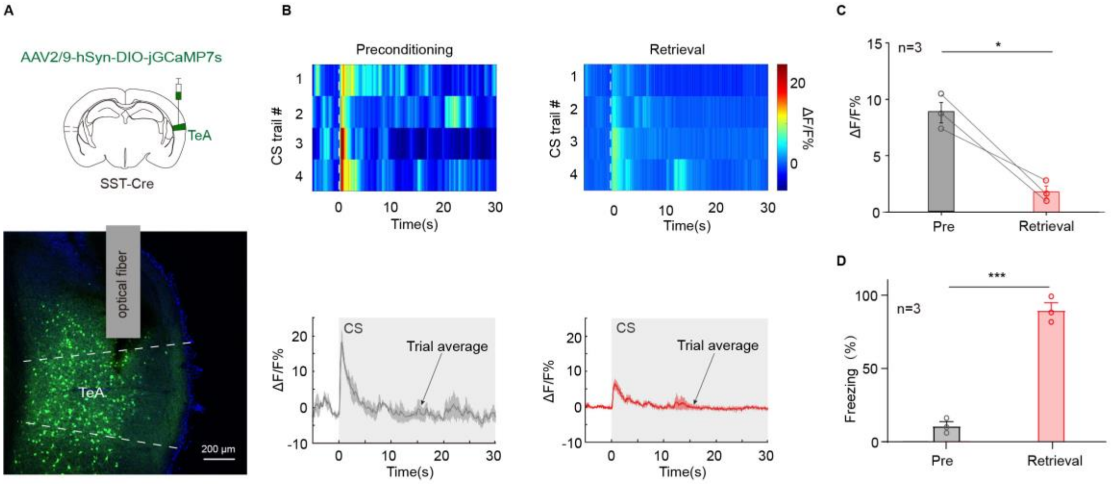

Supplementary figure legends:

Supplementary Fig.1：

Chemogenetic inhibition of TS neurons impaired noise auditory fear retrieval. (A) The injection sites of hM4D in TS with CNO i.p., and the freezing% during fear conditioning (B) and retrieval testing (C). hM4D- vs hM4D+, $t_{(10)}$=4.13, p= 2.03×10^{-3}, t test for CS.

Supplementary Fig.2：

Calcium signals in SST neurons are suppressed during fear retrieval. (A) Top, injection of AAV2/9-hSyn-DIO-jGCaMP7s in unilateral TeA of SST-Cre mice. Bottom, jGCaMP7s (green) expression at the injection site. (B) Heat maps (top) and mean Ca^{2+} traces± s.e.m. (shaded area) (bottom) of calcium signaling from SST neurons during preconditioning and retrieval test. (C-D) ΔF/F%(C) and CS-evoked freezing% (D) during the retrieval test for animals during preconditioning (gray) and retrieval (red) in (A). ΔF/F%: preconditioning vs retrieval, $t_{(2)}$= 5.43, p=0.0323, two-sided paired t test; Freezing%: preconditioning vs retrieval, $t_{(2)}$= -21.998, p=0.0021, two-sided paired t test.
